# Evolutionary Conserved Role of c-Jun-N-Terminal Kinase in CO_2_-Induced Epithelial Dysfunction

**DOI:** 10.1371/journal.pone.0046696

**Published:** 2012-10-08

**Authors:** István Vadász, Laura A. Dada, Arturo Briva, Iiro Taneli Helenius, Kfir Sharabi, Lynn C. Welch, Aileen M. Kelly, Benno A. Grzesik, G. R. Scott Budinger, Jing Liu, Werner Seeger, Greg J. Beitel, Yosef Gruenbaum, Jacob I. Sznajder

**Affiliations:** 1 Division of Pulmonary and Critical Care Medicine, Northwestern University, Chicago, Illinois, United States of America; 2 Department of Internal Medicine, Justus Liebig University, Universities of Giessen and Marburg Lung Center, Member of the German Center for Lung Research, Giessen, Germany; 3 Departamento de Fisiopatología, Facultad de Medicina, Universidad de la Republica, Montevideo, Uruguay; 4 Department of Molecular Biosciences, Northwestern University, Evanston, Illinois, United States of America; 5 Department of Genetics, Institute of Life Sciences, Hebrew University of Jerusalem, Givat Ram, Jerusalem, Israel; Comprehensive Pneumology Center, Germany

## Abstract

Elevated CO_2_ levels (hypercapnia) occur in patients with respiratory diseases and impair alveolar epithelial integrity, in part, by inhibiting Na,K-ATPase function. Here, we examined the role of c-Jun N-terminal kinase (JNK) in CO_2_ signaling in mammalian alveolar epithelial cells as well as in diptera, nematodes and rodent lungs. In alveolar epithelial cells, elevated CO_2_ levels rapidly induced activation of JNK leading to downregulation of Na,K-ATPase and alveolar epithelial dysfunction. Hypercapnia-induced activation of JNK required AMP-activated protein kinase (AMPK) and protein kinase C-ζ leading to subsequent phosphorylation of JNK at Ser-129. Importantly, elevated CO_2_ levels also caused a rapid and prominent activation of JNK in *Drosophila* S2 cells and in *C. elegans*. Paralleling the results with mammalian epithelial cells, RNAi against *Drosophila* JNK fully prevented CO_2_-induced downregulation of Na,K-ATPase in *Drosophila* S2 cells. The importance and specificity of JNK CO_2_ signaling was additionally demonstrated by the ability of mutations in the *C. elegans* JNK homologs, *jnk-1* and *kgb-2* to partially rescue the hypercapnia-induced fertility defects but not the pharyngeal pumping defects. Together, these data provide evidence that deleterious effects of hypercapnia are mediated by JNK which plays an evolutionary conserved, specific role in CO_2_ signaling in mammals, diptera and nematodes.

## Introduction

Hypercapnia is an emerging area of interest in the pathogenesis of pulmonary diseases including acute respiratory distress syndrome (ARDS) and chronic obstructive pulmonary disease (COPD). Under physiological conditions, the alveolar epithelium provides optimal gas exchange by minimizing fluid in the alveolar space through active vectorial Na^+^ transport driven in part by the Na,K-ATPase [1], [2], [3]. We have previously reported that hypercapnia, by downregulating the Na,K-ATPase, impairs alveolar fluid reabsorption (AFR), thereby leading to alveolar epithelial dysfunction [4], [5]; however, the mechanisms regulating the effects of hypercapnia have not been fully elucidated.

While chemoreception of CO_2_ in mammalian neurons have been described decades ago [6], only recently did it become clear that non-excitable mammalian cells are also capable of sensing, and responding to, changes in CO_2_ concentrations [7], [8], [9], [10], [11]. The c-Jun-N-terminal kinase (JNK), a member of the mitogen-activated protein kinase (MAPK) superfamily, plays a key role in cell adaptation to stress stimuli [12], [13], [14], [15]. The ubiquitously expressed isoform, JNK_1_ is phylogenetically highly conserved with orthologs in *Drosophila* and *C. elegans* [12], [13]. Activation of JNK requires its phosphorylation at the TPY motif (Thr-183 and Tyr-185) by MAPK kinases (MAPKK). Critically, phosphorylation of JNK_1_ at the Ser-129 residue by protein kinase C (PKC) has been shown to be required for maximal JNK induction [16], [17], [18].

Notably, not only mammalian cells sense and adapt to CO_2_ changes. For example, CO_2_ avoidance, which is mediated by specific neurons, has been demonstrated in both *C. elegans* and *Drosophila* [19], [20], [21]. Moreover, elevated CO_2_ levels also exhibit specific, non-neural effects in both *C. elegans* and *Drosophila*, which appear to be independent of any previously indentified stress adaptation pathways [22], [23]. Strikingly, as in mammals, elevated CO_2_ levels lead to rapid endocytosis of the Na,K-ATPase in *Drosophila* S2 cells by a yet unidentified mechanism [23]. We therefore hypothesized that the cellular responses to elevated CO_2_ levels might be mediated by JNK in mammals, *Drosophila* and *C. elegans* indicating that JNK may play a central, evolutionary conserved role in CO_2_ signaling and adaptation to hypercapnia.

## Results

### JNK Activation is Required for Hypercapnia-induced Decrease in Na,K-ATPase Plasma Membrane Abundance and AFR

**Figure 1:**
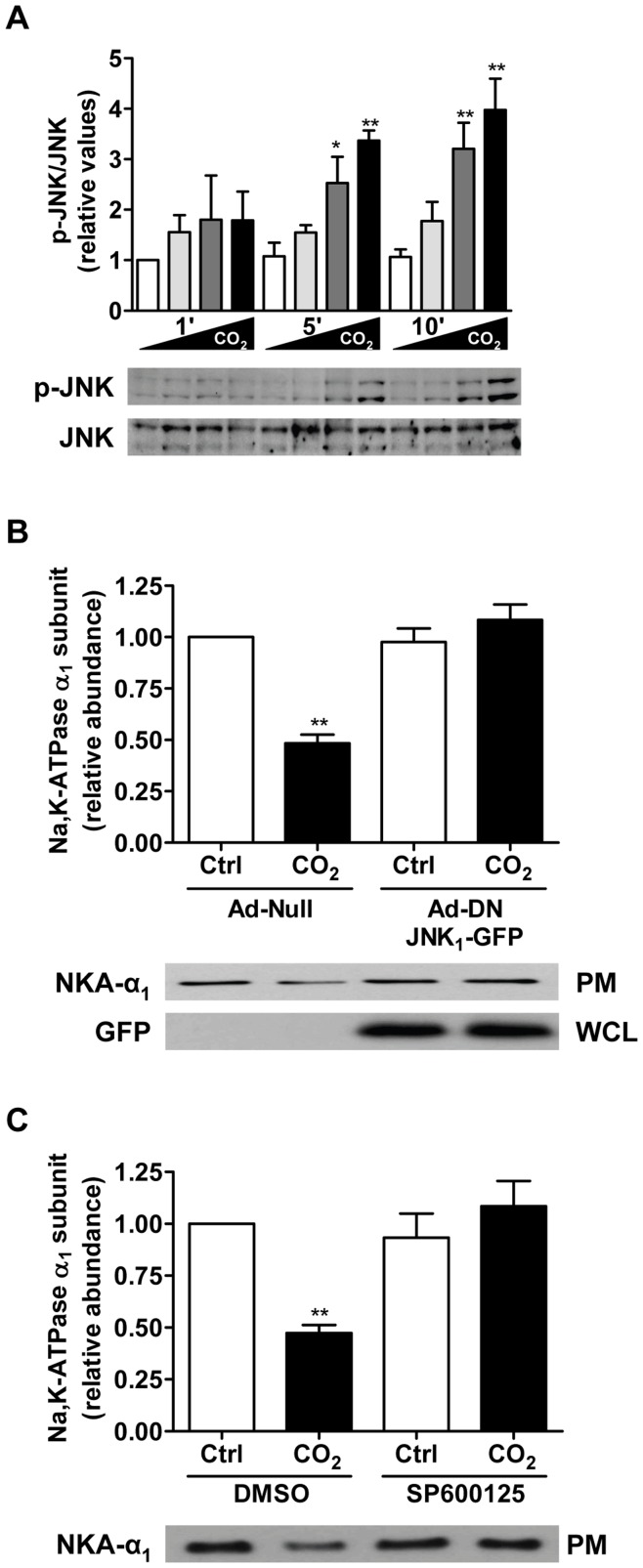
Activation of JNK by elevated CO_2_ levels is required for endocytosis of Na,K-ATPase in alveolar epithelial cells. (A) ATII cells were exposed to 40, 60, 80 or 120 mmHg CO**_2_** with a pH**_e_** of 7.4 (open, light grey-, grey- and black-closed bars, respectively) for 1, 5 and 10 min and phosphorylation of JNK at Thr-183/Tyr-185 (p-JNK) and total JNK (JNK) were measured by Western blot. (B) ATII cells were infected with a null adenovirus (Ad-Null) or GFP-tagged Ad-DN JNK1 and were exposed to 40 (open bars) or 120 (closed bars) mmHg CO**_2_** (pH**_e_** 7.4) for 30 min. Na,K-ATPase at the plasma membrane was determined by biotin-streptavidin pull down and subsequent Western blot analysis. (C) ATII cells were exposed to 40 (open bars) or 120 (closed bars) mmHg CO_2_ (pH_e_ 7.4) for 30 min in the presence or absence of SP600125 (5 µM, 30 min preincubation) and the amount of Na,K-ATPase protein at the plasma membrane was determined as in (B). Bars represent the mean ± SEM, n = 3. *, *p*<0.05, **, *p*<0.01. Representative Western blots of Na,K-ATPase α_1_-subunit at the plasma membrane and total protein abundance are shown. PM: plasma membrane, WCL: whole cell lysate.

To determine whether elevated CO_2_ activates JNK in the alveolar epithelium, we assessed JNK phosphorylation at residues Thr-183/Tyr-185, which reflects the activation status of JNK [13]. Exposure of rat alveolar epithelial type II (ATII) cells to elevated CO_2_ levels (60–120 mmHg at an extracellular pH (pH_e_) of 7.4) led to a concentration- and time-dependent phosphorylation of JNK (Figure 1A). Importantly, when ATII cells were exposed to extracellular acidosis, but normal CO_2_ levels (40 mmHg at a pH_e_ 7.2), JNK phosphorylation was not observed (Figure S1).

**Figure 2:**
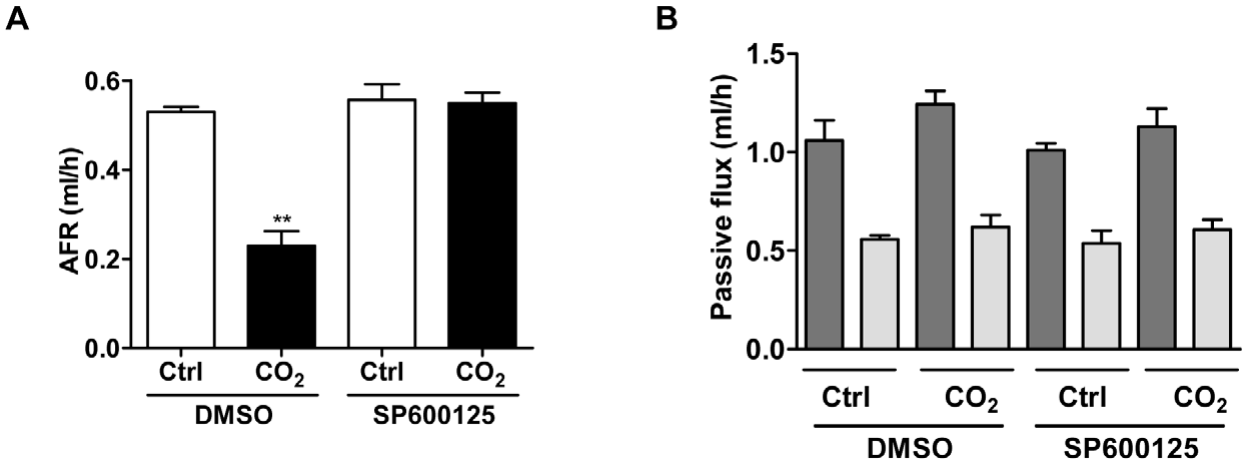
Activation of JNK by hypercapnia is required for inhibition of AFR in rat lungs. Isolated rat lungs were perfused for 1 h with 40 mmHg CO**_2_** (pHe 7.4; open bars) or with 60 mmHg CO**_2_** (pH**_e_** 7.2; solid bars) in the presence or absence of SP600125 (5 µM, 30 min preincubation) and (A) AFR and (B) and passive fluxes of ^22^Na^+^ (dark grey bars) and ^3^H-mannitol (light grey bars) were measured as described in the online supplementary material. Bars represent the mean ± SEM, n = 5, **, *p*<0.01. AFR: alveolar fluid reabsorption.

We have previously reported that hypercapnia leads to AFR impairment and promotes Na,K-ATPase endocytosis from the plasma membrane in ATII cells [4], [5]. Importantly, Na,K-ATPase endocytosis was prevented when ATII cells were infected with an adenovirus expressing a dominant-negative variant of JNK (DN-JNK_1_-GFP), while hypercapnia-induced Na,K-ATPase endocytosis was preserved in ATII cells infected with a null (Ad-null) virus (Figure 1B). Similarly, in the presence of the specific JNK inhibitor, SP600125 (Figure 1C) or siRNA against JNK (Figure S2), Na,K-ATPase endocytosis was prevented upon elevated CO_2_. Consistent with our findings in ATII cells, CO_2_-induced impairment in AFR was prevented in rat lungs pretreated with SP600125 (Figure 2A) without effecting passive movement of small solutes (Figure 2B), suggesting that JNK activation was required for both hypercapnia-induced downregulation of the Na,K-ATPase in the alveolar epithelium and impairment of AFR.

### Activation of AMPK and PKC-ζ are Necessary to Stimulate JNK Upon Hypercapnia in Alveolar Epithelial Cells

**Figure 3:**
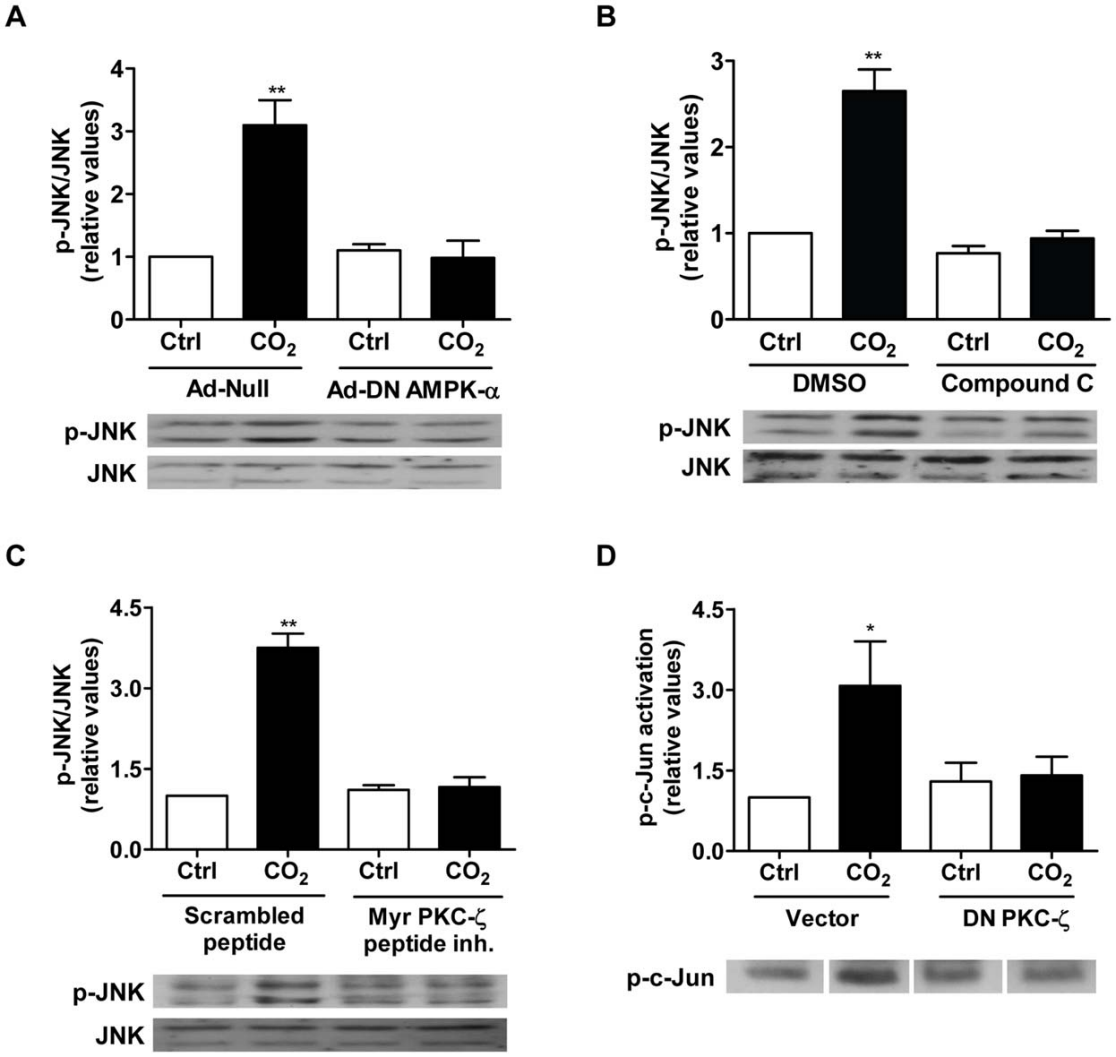
CO_2_-induced activation of JNK is dependent on AMPK and PKC-ζ. (A) ATII cells were infected with Ad-Null or HA-tagged Ad-DN AMPK-α**_1_** and were exposed 24 h later to 40 (open bars) or 120 (closed bars) mmHg CO**_2_** (pH**_e_** 7.4) for 10 min and JNK activation was assessed. (B) ATII cells were exposed to 40 (open bars) or 120 (closed bars) mmHg CO_2_ (pH_e_ 7.4) for 10 min in the presence or absence of Compound C (20 µM, 30 min preincubation). (C) ATII cells were exposed to 40 (open bars) or 120 (closed bars) mmHg CO**_2_** (pH**_e_** 7.4) for 10 min in the presence of a myristoylated peptide inhibitor of PKC-ζ (15 µM, 30 min preincubation) or a scrambled peptide. Activation of JNK was determined by Western blot as described above. (D) A549 cells expressing an empty vector or DN PKC-ζ were grown confluent in the presence of G418 after which cells were exposed to 40 (open bars) or 120 (closed bars) mmHg CO**_2_** (pH**_e_** 7.4) for 10 min. JNK was immunoprecipitated and incubated with c-Jun and p-c-Jun was measured by Western blot. Bars represent the mean ± SEM, n ≥3. *, *p*<0.05; **, *p*<0.01. Representative Western blots of p-JNK and total JNK (A-C) or p-c-Jun (D) are shown.

We have previously demonstrated that the AMP-activated protein kinase (AMPK) is an important element of CO_2_ sensing [5]. Since hypercapnia rapidly activates AMPK (within 1 min), we next examined whether JNK was a downstream target of AMPK. Indeed, JNK phosphorylation, induced by exposure of ATII cells to elevated CO_2_ levels for 10 min, was prevented when ATII cells were infected with an adenovirus expressing a dominant-negative variant of AMPK-α_1_ (DN-AMPK-α_1_; Figure 3A). Similarly, JNK phosphorylation was also inhibited by the AMPK inhibitor Compound C (Figure 3B), suggesting that AMPK acts upstream of JNK in the CO_2_-induced signaling cascade. Furthermore, treatment of ATII cells with AICAR, a chemical activator of AMPK, led to JNK phosphorylation (Figure S3); thus, AMPK activation was sufficient to stimulate JNK.

**Figure 4:**
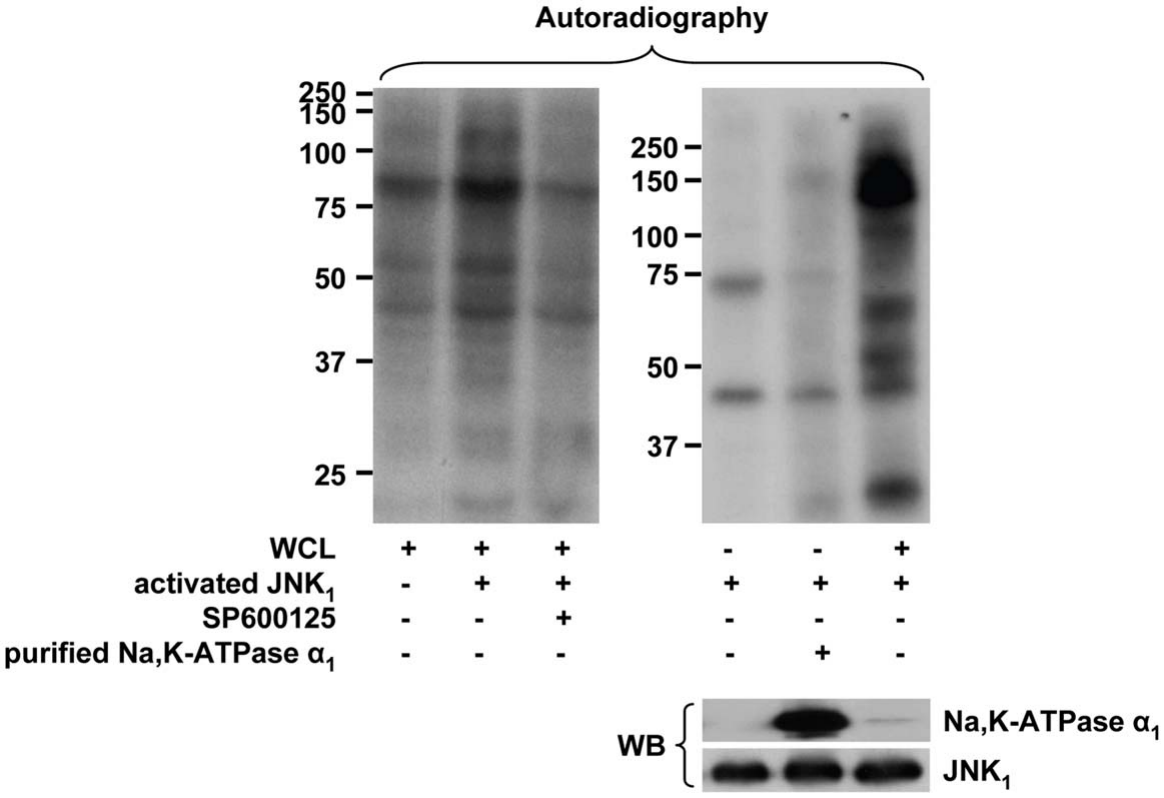
JNK does not phosphorylate the Na,K-ATPase α-subunit. An *in vitro* JNK kinase was performed in the presence and absence of ATII whole cell lysate (WCL), purified Na,K-ATPase α_1_-subunit or the specific JNK inhibitor SP600125 or as described in the “Supplemental methods”. Representative autoradiographs (upper panels) and a western blot (lower panel) of Na,K-ATPase α_1_ and JNK_1_ corresponding to the left autoradiograph are shown.

We and others have previously identified PKC-ζ as an important regulator of Na,K-ATPase [24], [25], which is downstream of AMPK in the hypercapnia-induced signaling cascade [5]. Therefore, we next asked whether activation of JNK was regulated by PKC-ζ. Pretreatment of ATII cells with a myristoylated peptide inhibitor of PKC-ζ completely prevented the CO_2_-induced JNK activation (Figure 3C). Similar results were obtained in A549 cells overexpressing a dominant negative variant of PKC-ζ (DN PKC-ζ, Figure 3D), suggesting that PKC-ζ acts upstream of JNK. Moreover, preincubation of ATII cells with a high (but not with a low) dose of the PKC inhibitor bisindolylmaleimide I prevented the CO_2_-induced JNK activation (Figure S4A), further confirming that an atypical (as opposed to classical and novel) isoform of PKC was necessary for the CO_2_-induced JNK phosphorylation. In line with these findings, phorbol 12-myristate 13-acetate and Gö 6976, inhibitors of classical PKCs, did not affect activation of JNK upon hypercapnia (Figure S4B). Interestingly, as opposed to PKC-ζ [4], JNK did not phosphorylate the Na,K-ATPase (Figure 4), suggesting that JNK may regulate the process of Na,K-ATPase trafficking.

### Phosphorylation of JNK at Ser-129 by PKC-ζ Leads to JNK Activation and Na,K-ATPase Endocytosis during Exposure to Elevated CO_2_

**Figure 5:**
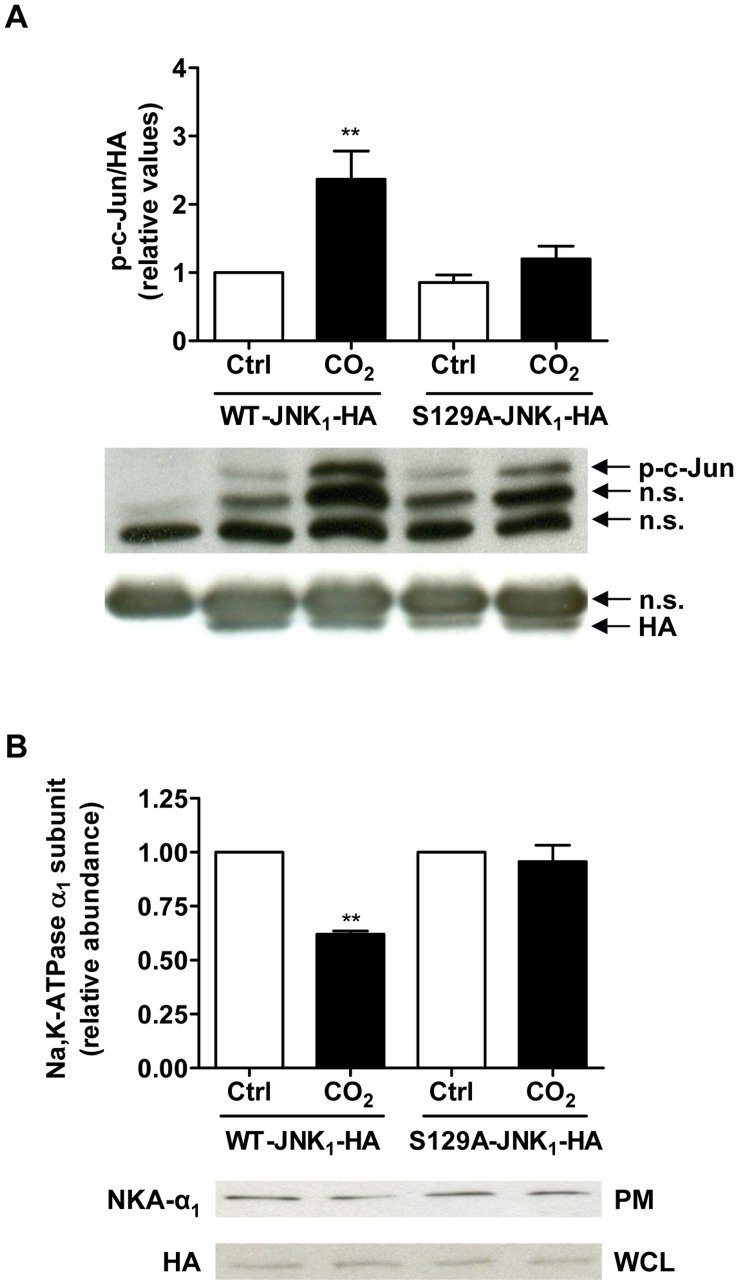
CO_2_-induced activation of JNK is dependent on Ser-129 phosphorylation downstream of PKC-ζ. (A) A549 cells transfected with a wild-type JNK (WT-JNK**_1_**-HA) or with a mutant variant in which the Ser-129 residue was mutated to alanine (S129A-JNK**_1_**-HA) were exposed to 40 (open bars) or 120 (closed bars) mmHg CO**_2_** (pH**_e_** 7.4) for 10 min. JNK was immunoprecipitated and incubated with c-Jun and p-c-Jun was measured by Western blot. n.s.: non-specific bands. (B) A549 cells transfected with WT-JNK**_1_**-HA or S129A-JNK**_1_**-HA were exposed to 40 (open bars) or 120 (closed bars) mmHg CO**_2_** (pH**_e_** 7.4) for 30 min. The amount of Na,K-ATPase protein at the plasma membrane was determined by biotinylation as described above. PM: plasma membrane, WCL: whole cell lysate. Values are expressed as mean ± SEM, n = 3. **, *p*<0.01.

PKC-α, -β and -δ have been previously shown to phosphorylate JNK at its Ser-129 residue, thereby augmenting its activation by MAPKK [16], [17], [18]. Therefore, we next asked whether the PKC-ζ-mediated JNK activation upon hypercapnia was a consequence of JNK phosphorylation at Ser-129 by PKC-ζ. Immunokinase assays that used c-Jun as a substrate showed an increase in JNK activity after exposure to hypercapnia in A549 cells transfected with WT-JNK_1_-HA. In contrast, overexpression of a mutant variant of JNK_1_ in which Ser-129 was mutated to alanine (S129A-JNK_1_-HA) prevented the hypercapnia-induced phosphorylation of the JNK downstream target c-Jun (Figure 5A), suggesting that the Ser-129 residue may serve as PKC phospho-acceptor site. Furthermore, overexpression of S129A-JNK_1_-HA (as opposed to wild-type JNK) prevented endocytosis of the Na,K-ATPase during hypercapnia (Figure 5B), suggesting that phosphorylation of JNK at Ser-129 by PKC-ζ is required for JNK activation which in turn drives endocytosis of the Na,K-ATPase.

### JNK is Required for CO_2_-induced Na,K-ATPase Endocytosis in *Drosophila* S2 Cells and Mediates some, but not all, CO_2_ Responses in *C. elegans*

**Figure 6:**
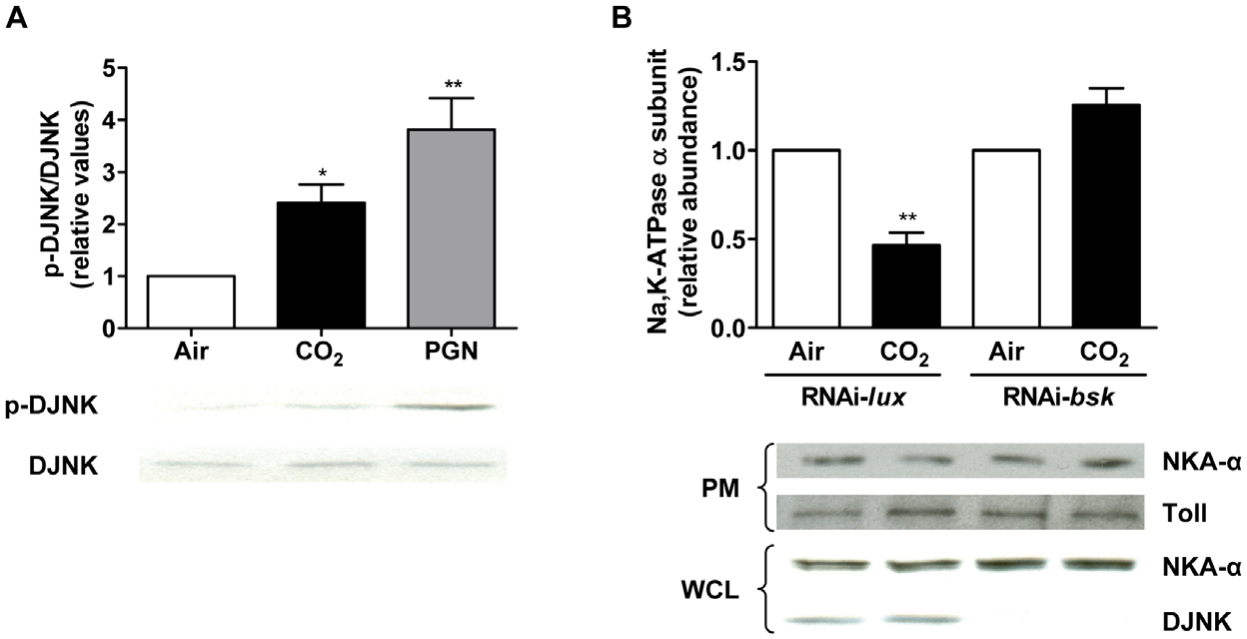
CO_2_-induced downregulation of Na,K-ATPase in *Drosophila* is mediated by JNK. (A) *Drosophila* S2 cells were allowed to attach to 6-well plates, were exposed to air (open bars) or 120 mmHg CO**_2_** (pHe 7.4, closed bars) for 30 min and phosphorylation of DJNK at Thr-183/Tyr-185 (p-DJNK) and total DJNK were measured by Western blot. *E. coli* peptidoglycan (PGN; 25 mg/ml, 15 min) was used as positive control. (B) *Drosophila* S2 cells were grown for 5 days after incubation with RNAi against DJNK/basket (RNAi-*bsk*) or a non-relevant RNAi (RNAi-*lux*). Thereafter cells were exposed to air (open bars) or 120 mmHg CO**_2_** (pH**_e_** 7.4, closed bars) for 1 h and membrane abundance of the *Drosophila* Na,K-ATPase was assessed by cell surface biotinylation, Toll served as loading control. Values are expressed as mean ± SEM, n = 3, *, *p*<0.05; **, *p*<0.01. PM: plasma membrane, WCL: whole cell lysate.

Since CO_2_ is a metabolite of all aerobic respiration, we reasoned that some of the mechanisms by which cells respond to CO_2_ accumulation may be evolutionary conserved. We therefore next investigated whether JNK might mediate CO_2_ responses in *Drosophila*. Interestingly, exposure of *Drosophila* S2 cells to elevated CO_2_ for 30 min resulted in a significant activation of *Drosophila* JNK (DJNK, also known as Basket; Figure 6A). Furthermore, RNA knockdown of the *basket* gene (*bsk*) completely prevented the CO_2_-induced Na,K-ATPase endocytosis (Figure 6B). Thus, JNK is not only critically involved in CO_2_ signaling in mammalian cells but also in diptera.

**Figure 7:**
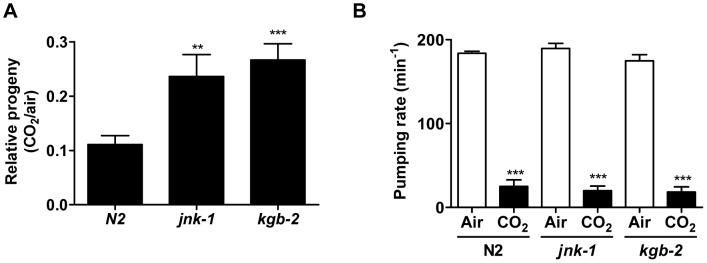
JNK mediates CO_2_-induced inhibition of fertility but not impaired pharyngeal pumping rate in *C. elegans*. (A) Gravid *C. elegans* worms of genotype wild type (N2) or the null mutants *jnk-1 (gk7)* or *kgb-2(gk361)* were allowed to lay eggs for 6 hours at 20°C in either 19% CO**_2_** or in control air condition and the number of eggs laid in 19% CO**_2_** was normalized to the number of eggs laid in air condition. (B) Wild type and mutant worms were grown in normal air conditions until their first day of adulthood, exposed to air (open bars) or 19% CO**_2_** (closed bars) for 10 min and pharyngeal pumping rate was scored. Values are expressed as mean ± SEM, n = 30, **, *p*<0.01; ***, *p*<0.001.

To further test for conservation of the role of JNK in CO_2_ responses, we investigated if JNK was required for the CO_2_-induced reductions in fertility and pharyngeal pumping in *C. elegans* that we had previously observed [22]. Deletion of the JNK homologs jnk-1 or kgb-2 significantly rescued the hypercapnia-induced impairment of fertility (Figure 7A). In contrast, pharyngeal pumping rate, which was markedly decreased upon exposure to elevated CO_2_, was not affected by *jnk-1(gk7)* or *kgb-2(gk361)* null mutations (Figure 7B), highlighting the specificity of JNK action upon hypercapnia.

## Discussion

**Figure 8:**
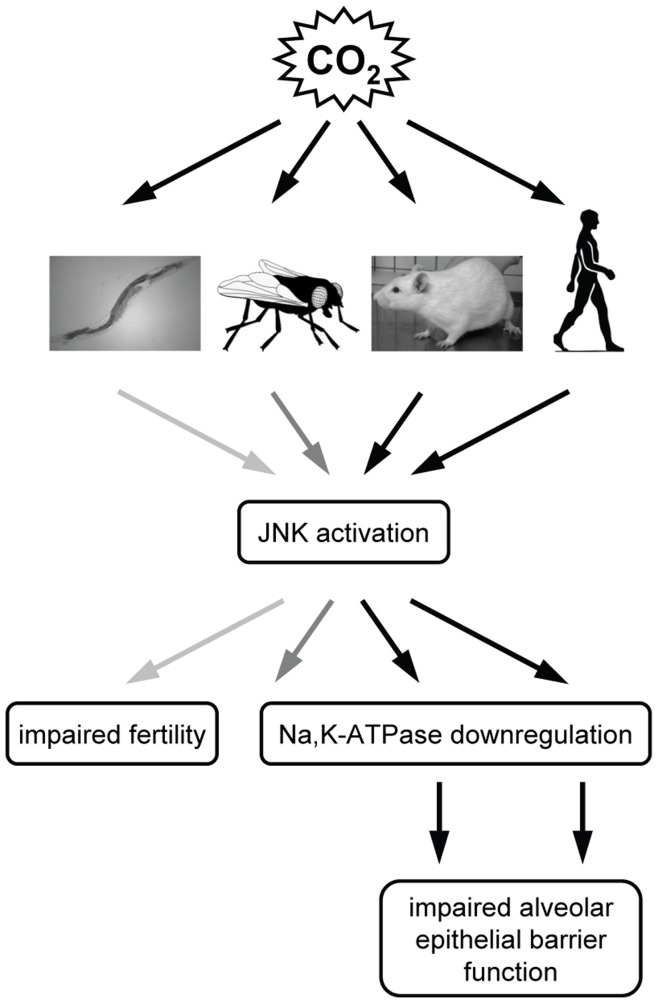
Schematic representation of the evolutionary conserved CO_2_-induced effects. Elevated CO_2_ levels rapidly activate JNK in *C. elegans*, *Drosophila*, rat lungs and human alveolar epithelial cells. In *C. elegans* (light grey arrows) the CO_2_-induced JNK activation leads to impaired fertility. In Drosophila (dark gray arrows) and in the rat and human alveolar epithelium (black arrows) the hypercapnia-induced JNK activation decreases Na,K-ATPase membrane stability leading to impaired alveolar epithelial barrier function in mammals.

CO_2_ is a metabolite that has been produced by cells since aerobic respiration evolved over 2 billion years ago. One therefore would expect that some conservation of the mechanisms that cells use to respond to accumulation of CO_2_ may exist. Here we provide evidence that JNK activation is an evolutionary conserved mediator of CO_2_ responses (Figure 8).

The role of hypercapnia in the pathogenesis of pulmonary diseases associated with alveolar hypoventilation, such as ARDS and COPD, remains incompletely understood. Particularly, the mechanisms by which the alveolar epithelium, the primary site of gas exchange and thus CO_2_ elimination, senses and adapts to changes in CO_2_ levels are largely unknown. We have previously demonstrated that functional integrity of the alveolar epithelium is rapidly impaired by changes in CO_2_ concentrations [4], [26], [27]. In this study, we report that clinically relevant elevated CO_2_ levels led to rapid activation of JNK in alveolar epithelial cells. Importantly, and in line with our previous findings showing that the deleterious effects of hypercapnia on the alveolar epithelium were independent of changes in extra- or intracellular pH [4], the CO_2_-induced activation of JNK was also independent of pH. Critically, our data reveals that JNK activation was required for the hypercapnia-induced downregulation of the Na,K-ATPase in the alveolar epithelium and impairment of AFR. Previously, JNK has been shown to have a key role in promoting cellular adaptation to various stress stimuli and has recently emerged as a novel regulator of epithelial transport function [28], [29] and Na,K-ATPase activity [30], [31], but JNK has not been implicated in CO_2_ responses.

Why elevated CO_2_ concentrations lead to downregulation of the Na,K-ATPase has not been fully elucidated. It is well known, that the Na,K-ATPase accounts for approximately 40% of cellular energy expenditure [2] and hypercapnia suppresses select physiological functions that are known to be metabolically demanding [4], [22], [23]. Thus, it is possible that elevated levels of CO_2_ signal excessive metabolic load. Consistent with this hypothesis, we have previously reported that AMPK, a metabolic sensor that downregulates energy-consuming events, is an important element of CO_2_ sensing [5], [8]. Moreover, we and others have previously identified PKC-ζ as an important regulator of Na,K-ATPase [24], , which is downstream of AMPK in the hypercapnia-induced signaling cascade [5]. Therefore, we next asked whether activation of JNK was regulated by AMPK and PKC-ζ. By applying genetic and pharmacological approaches, we found that both AMPK and PKC-ζ act upstream of JNK in the CO_2_-induced signaling cascade in alveolar epithelial cells.

Recently, the extracellular signal-regulated kinase (ERK), another member of the MAPK superfamily, has been also shown to play an important role in CO_2_ signaling [33]. However, activation of ERK is extremely fast, peaking within seconds after CO_2_ exposure and returning to baseline levels within 5 min [33] as opposed to JNK activation which occurs after 5–10 min. Interestingly, and in contrast to the role of JNK during CO_2_ signaling, ERK was found to be upstream of AMPK playing a modulatory role in the AMPK-mediated sensing of CO_2_. Thus these two members of the MAPK superfamily are both involved but play distinct roles in CO_2_ sensing and signaling.

Once activated, PKC-ζ phosphorylates the Na,K-ATPase α_1_-subunit at the Ser-11 or Ser-18 (depending on the species) thereby promoting its endocytosis [4], [25], [34]. Since mutation of Ser-18 of the Na,K-ATPase α_1_-subunit prevents the CO_2_-induced endocytosis of the Na,K-ATPase upon hypercapnia [4] one might ask how JNK (which is apparently downstream of PKC-ζ) can be involved in this process. Since JNK did not directly phosphorylate the Na,K-ATPase, it is possible that JNK regulates the process of Na,K-ATPase trafficking. Indeed, various reports described that JNK may modulate cytoskeletal rearrangement and function of molecular motors involved in trafficking of membrane proteins [35], [36], [37]. Also, we cannot fully exclude the involvement of other intermediates in the CO_2_-induced signaling pattern. These possibilities are currently under investigation in our laboratory.

It is well established that JNK activation requires its phosphorylation at the TPY motif by MAPKK [12]. Interestingly, classical PKCs and PKC-δ, a novel PKC isoform, have been shown to be required for JNK induction by diverse stimuli including cytokines and UV-irradiation by phosphorylating JNK at Ser-129, thereby further augmenting its activation [16], [17], [18]. In the current study we found that phosphorylation of JNK at the Ser-129 residue is required for JNK activation and Na,K-ATPase endocytosis upon hypercapnia and that this residue may serve as PKC-ζ phospho-acceptor site.

Recently, it has become increasingly clear that cells and organisms respond to CO_2_ and that some of those responses are highly similar [7]. For example, both mammalian alveolar epithelial cells and *Drosophila* S2 cells reduce their surface Na,K-ATPase levels in elevated CO_2_ conditions [23]. However, it has not been clear whether the similar cellular responses were controlled by conserved intracellular processes. Remarkably, blocking JNK signaling prevented Na,K-ATPase endocytosis in both mammalian and *Drosophila* cells, strongly supporting the hypothesis that at least some responses to CO_2_ are evolutionary conserved. Furthermore, downregulation of the *C. elegans* JNK homologs jnk-1 or kgb-2 significantly rescued the reduction in fertility but not the pharyngeal pumping rate defects caused by elevated CO_2_ levels, suggesting the existence of multiple pathways that can mediate CO_2_ responses and highlighting the specificity of JNK action.

In summary, we provide evidence that JNK activation is an evolutionary conserved mediator of CO_2_ responses in mammals, *Drosophila melanogaster* and *C. elegans*. Further, we identify mammalian PKC-ζ as a novel upstream kinase responsible for JNK phosphorylation at Ser-129, leading to downregulation of the Na,K-ATPase and thus alveolar epithelial dysfunction.

## Materials and Methods

The CO_2_ media, cell surface biotinylation and isolated-perfused lung preparation have been described in detail previously [4], [5], [25], [38]. A brief description of methodologies and reagents is provided in the Supplemental Material.

### Ethics Statement

Animals were handled according to National Institutes of Health guidelines and the experimental protocol for the use of rats (2010–2177) was approved by the Animal Care and Use Committee at Northwestern University.

### Adenoviral Infection of ATII Cells

*Day* 2 ATII cells, plated on 60-mm cell culture dishes were incubated with null adenovirus (Ad-Null, 20 pfu/cell) or with adenovirus expressing a dominant-negative, JNK_1_ tagged with GFP (Ad-DN JNK_1_-GFP) as previously described [39], or carrying a dominant-negative, kinase dead (K45R) variant of the AMPK-α_1_-subunit (Ad-DN AMPK-α_1_, (a generous gift from Prof. Lee A. Witters, Dartmouth College; 20 pfu/cell) [40] in 500 µl DMEM. After an incubation period of 2–4 h, 1.5 ml of DMEM supplemented with 10% fetal bovine serum, 100 U/ml penicillin, 100 µg/ml streptomycin was added to the cell culture plates and experiments were performed 24 h later.

### Transient Transfection of A549 Cells

The expression vectors encoding an HA-tagged wild-type JNK_1_ (WT-JNK_1_-HA) and a mutant JNK_1_ variant in which Ser-129 was replaced by an alanine (S129A-JNK_1_-HA) have been described previously [16]. A549 cells (American Type Culture Collection; Manassas, VA) were plated in 60-mm cell culture dishes and transfected with 4 µg DNA using Lipofectamine 2000 (Invitrogen, Carlsbad, CA) as recommended by the manufacturer, and experiments were performed 24 h later.

### RNA Knockdown and Na,K-ATPase Membrane Abundance in *Drosophila* S2 Cells

For RNA knockdown, 5×10^5^ *Drosophila* S2 cells (Invitrogen, Carlsbad, CA) were incubated with 7.5 µg double-stranded RNA for 30 min in serum-free medium in 6-well plates, and thereafter grown in 10% FBS-containing medium for 5 days. CO_2_ treatments were performed at 15% CO_2_ using pre-equilibrated media buffered to pH equal to air condition, with S2 cells attached to 6-well plates. Cell surface biotinylation was performed as previously described after 1 h in 15% CO_2_ [23].

### Progeny Number and Pharyngeal Pumping Measurements in *C. elegans*

Gravid worms of wild type (N2), *jnk-1(gk7)* and *kgb-2(gk361)* null mutations were allowed to lay eggs for 6 hours at 20°C in either 19% CO_2_ or in control air condition. After 6 hours adult worms were removed, progeny was scored as described previously [22]. In experiments assessing pharyngeal pumping, animals were grown in normal air conditions until their first day of adulthood. Pumping rate was scored after 10 minutes exposure to air or 19% CO_2_.

### Data Analysis

Data are expressed as mean ± SEM. Data were compared using analysis of variance adjusted for multiple comparisons with the Dunnet test. When comparisons were performed between two groups of values, significance was evaluated by Student’s test. A *p* value<0.05 was considered significant.

## Supporting Information

Figure S1 **ATII cells were exposed to 40 mmHg CO_2_ with a pH_e_ of 7.4 or to 40 mmHg CO_2_ with a pH_e_ of 7.2 for 10 min and the phosphorylation of JNK at Thr-183/Tyr-185 (p-JNK) and the total amount of JNK (JNK) was measured by Western blot analysis.** Top: Graph represents the p-JNK/JNK ratio. Values are expressed as mean ± SEM, n = 3. Bottom: Representative Western blots of p-JNK and total JNK. pH_e_: extracellular pH.
(TIF)

Figure S2 (A) A549 cells were transfected with siRNA against JNK_1_ (siRNA - JNK_1_) or scrambled siRNA (scr siRNA) as described in the Supplemental methods. Twenty four hours after transfection cells were exposed to 40 or 120 mmHg CO**_2_** (pH**_e_** 7.4) for 10 min. Representative Western blots of p-c-Jun, JNK_1_ and JNK_2_ as well as actin (loading control) from A549 whole cell lysates (WCL) are shown. (B) Twenty four hours after transfection A549 were exposed to 40 (open bars) or 120 (closed bars) mmHg CO**_2_** (pH**_e_** 7.4) for 30 min. Na,K-ATPase at the plasma membrane was determined by biotin-streptavidin pull down and subsequent Western blot analysis. Bars represent the mean ± SEM, n = 3, **, *p*<0.01. Representative Western blots of Na,K-ATPase α_1_-subunit and E-cadherin (E-cad) at the plasma membrane (PM) are shown. n.s.: non-specific band.
(TIF)

Figure S3 **ATII cells were treated with 2 mM AICAR or its vehicle for 30 or 60 min and phosphorylation of AMPK, acetyl-CoA carboxylase (ACC) and JNK (p-AMPK, p-ACC and p-JNK, respectively) and the amount of total AMPK and JNK were determined by Western blot. Representative Western blots are shown.**
(TIF)

Figure S4 (A) ATII cells were exposed to 40 (open bars) or 120 (closed bars) mmHg CO_2_ (pH_e_ 7.4) for 10 min in the presence or absence of bisindolylmaleimide I (Bis; 1 or 10 µM, 30 min preincubation). p-JNK and total JNK were determined by Western blot. Graph represents the p-JNK/JNK ratio, values are expressed as mean ± SEM, n = 3. **, *p*<0.01. Representative Western blots of p-JNK and total JNK are shown. (B) ATII cells were exposed to 40 (open bars) or 120 (closed bars) mmHg CO_2_ (pH_e_ 7.4) for 10 min in the presence or absence of Gö6976 (1 µM, 30 min preincubation) or PMA (25 µM, 24 h preincubation). p-JNK and total JNK were determined by Western blot. Graph represents the p-JNK/JNK ratio, values are expressed as mean ± SEM, n = 3. **, *p*<0.01. Representative Western blots of p-JNK and total JNK are shown.
(TIF)

Methods S1 **Supporting Methods.**
(PDF)
