# Supplementary figures and images for: Evolutionary Conserved Role of c-Jun-N-Terminal Kinase in CO2-Induced Epithelial Dysfunction
Source: PLoS One. 2012 Oct 8;7(10):e46696. doi: 10.1371/journal.pone.0046696 (PMC3466313; doi:10.1371/journal.pone.0046696)

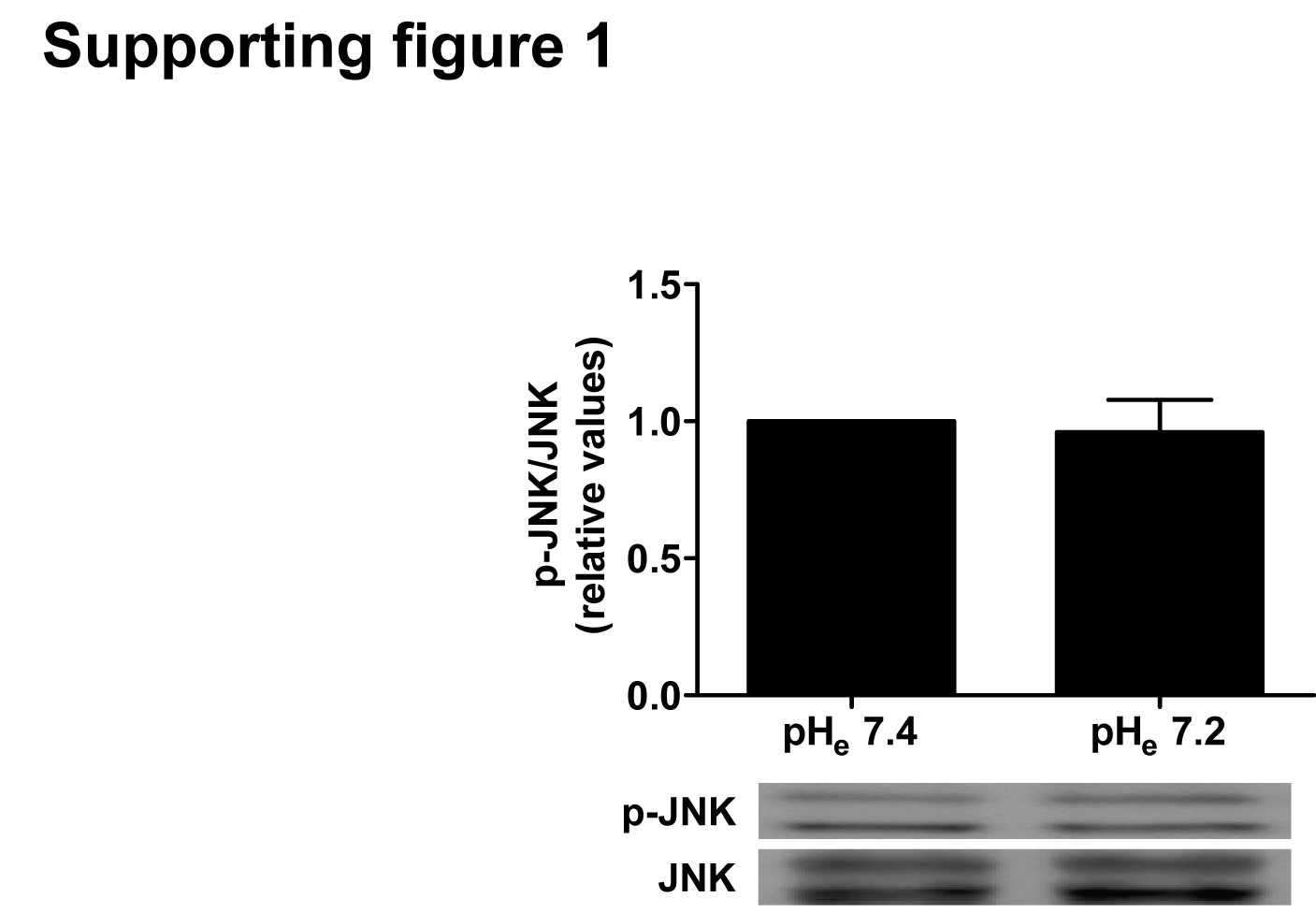

Supplement: Figure S1 — ATII cells were exposed to 40 mmHg CO2 with a pHe of 7.4 or to 40 mmHg CO2 with a pHe of 7.2 for 10 min and the phosphorylation of JNK at Thr-183/Tyr-185 (p-JNK) and the total amount of JNK (JNK) was measured by Western blot analysis. Top: Graph represents the p-JNK/JNK ratio. Values are expressed as mean ± SEM, n = 3. Bottom: Representative Western blots of p-JNK and total JNK. pHe: extracellular pH. (TIF) [file pone.0046696.s001.tif]

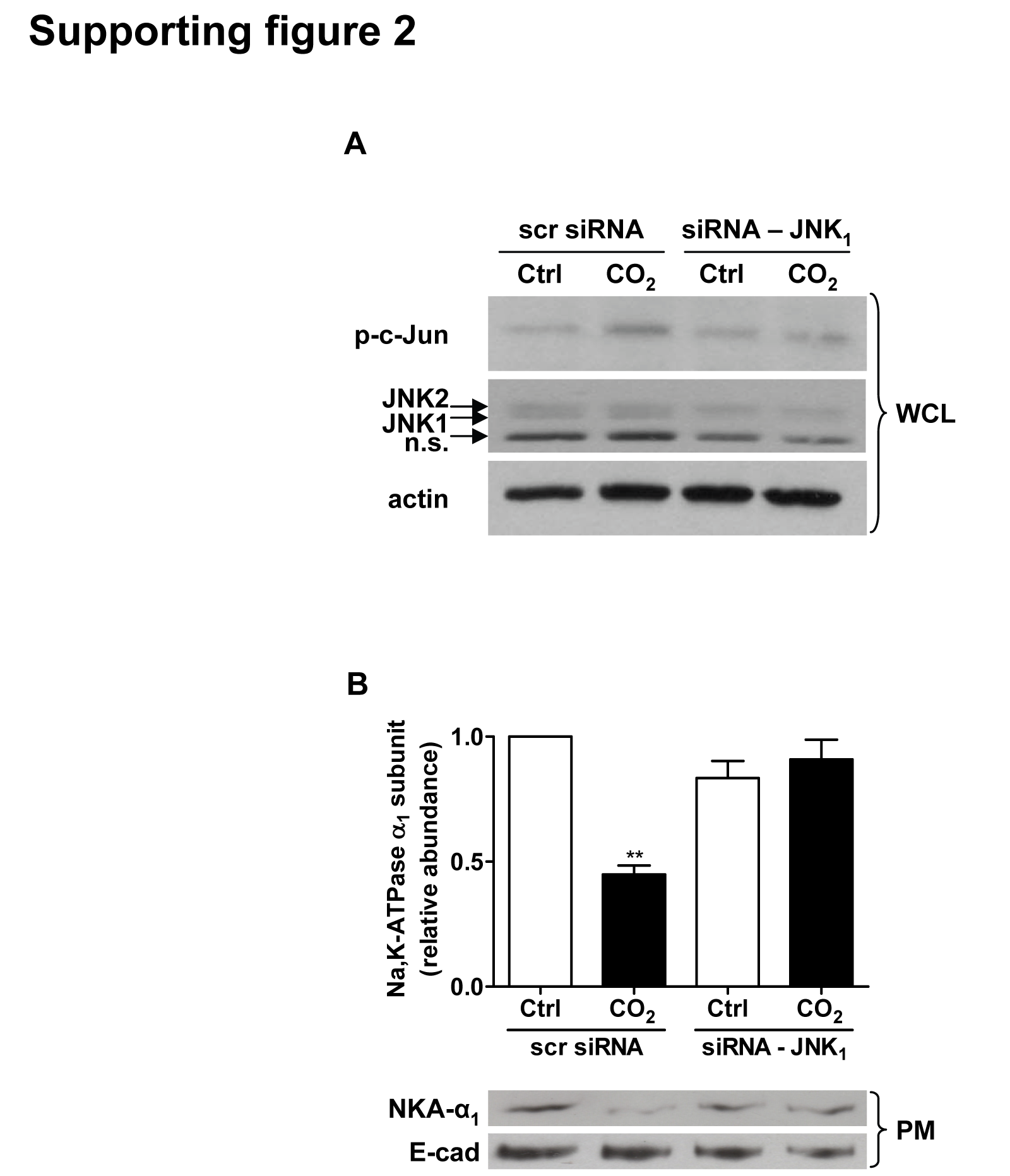

Supplement: Figure S2 — (A) A549 cells were transfected with siRNA against JNK1 (siRNA - JNK1) or scrambled siRNA (scr siRNA) as described in the Supplemental methods. Twenty four hours after transfection cells were exposed to 40 or 120 mmHg CO2 (pHe 7.4) for 10 min. Representative Western blots of p-c-Jun, JNK1 and JNK2 as well as actin (loading control) from A549 whole cell lysates (WCL) are shown. (B) Twenty four hours after transfection A549 were exposed to 40 (open bars) or 120 (closed bars) mmHg CO2 (pHe 7.4) for 30 min. Na,K-ATPase at the plasma membrane was determined by biotin-streptavidin pull down and subsequent Western blot analysis. Bars represent the mean ± SEM, n = 3, **, p<0.01. Representative Western blots of Na,K-ATPase α1-subunit and E-cadherin (E-cad) at the plasma membrane (PM) are shown. n.s.: non-specific band. (TIF) [file pone.0046696.s002.tif]

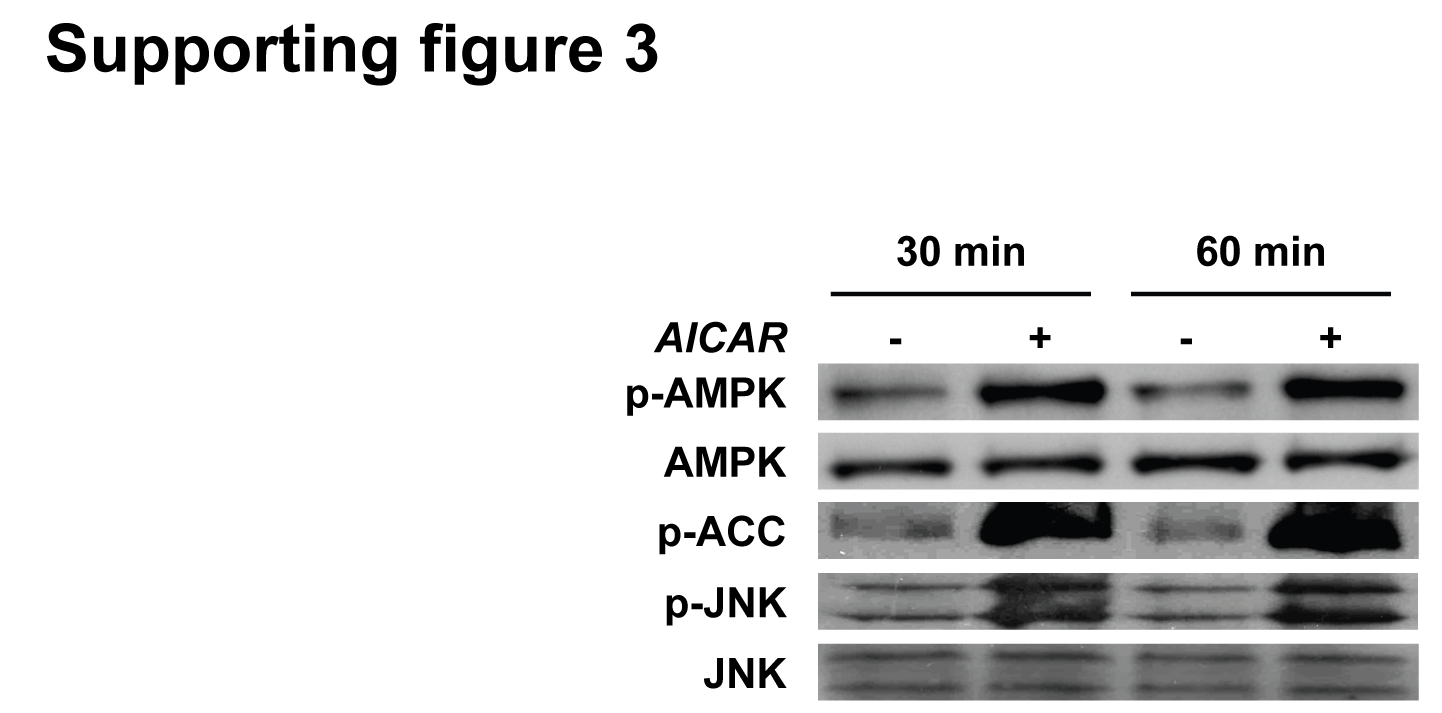

Supplement: Figure S3 — ATII cells were treated with 2 mM AICAR or its vehicle for 30 or 60 min and phosphorylation of AMPK, acetyl-CoA carboxylase (ACC) and JNK (p-AMPK, p-ACC and p-JNK, respectively) and the amount of total AMPK and JNK were determined by Western blot. Representative Western blots are shown. (TIF) [file pone.0046696.s003.tif]

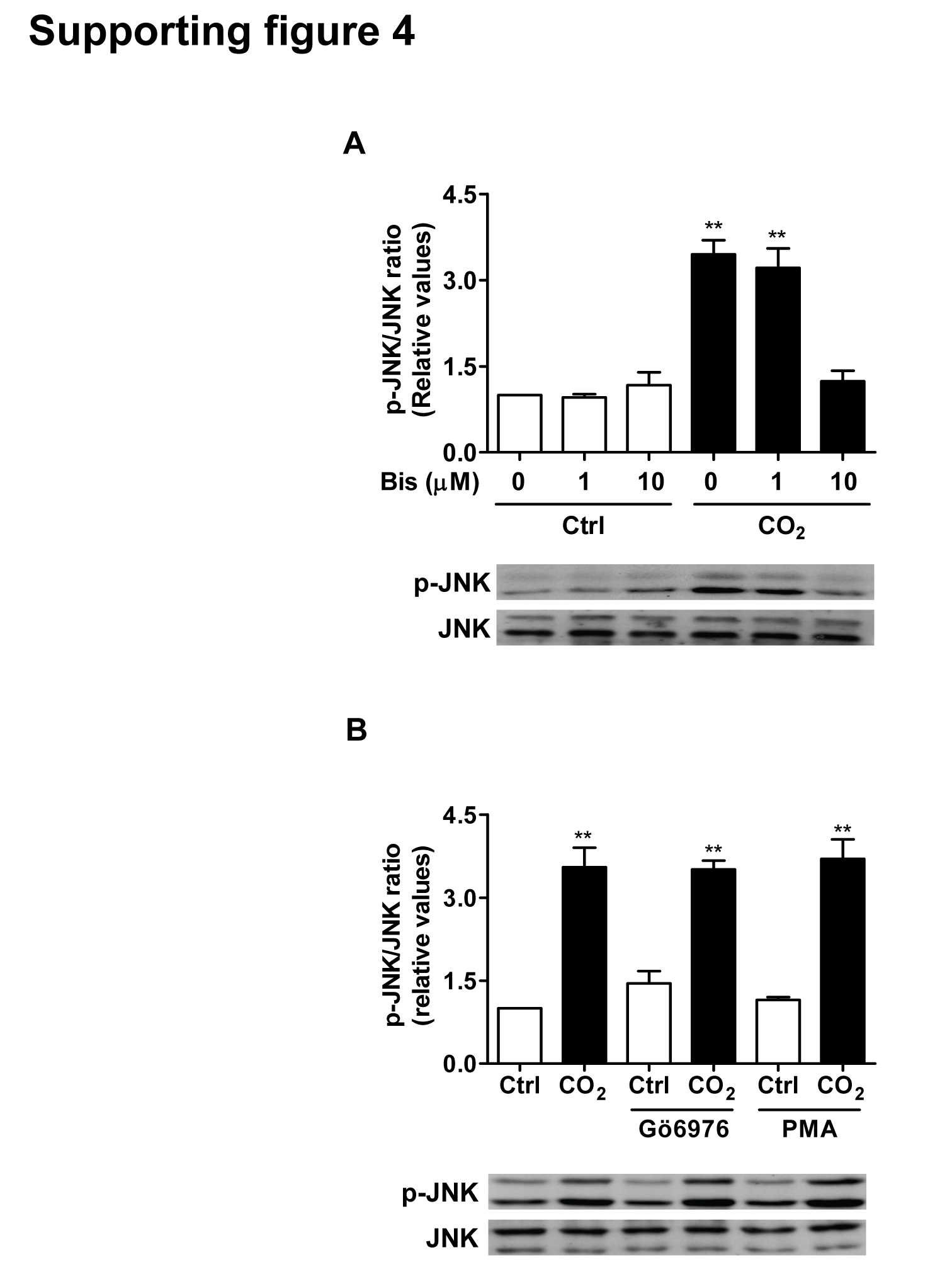

Supplement: Figure S4 — (A) ATII cells were exposed to 40 (open bars) or 120 (closed bars) mmHg CO2 (pHe 7.4) for 10 min in the presence or absence of bisindolylmaleimide I (Bis; 1 or 10 µM, 30 min preincubation). p-JNK and total JNK were determined by Western blot. Graph represents the p-JNK/JNK ratio, values are expressed as mean ± SEM, n = 3. **, p<0.01. Representative Western blots of p-JNK and total JNK are shown. (B) ATII cells were exposed to 40 (open bars) or 120 (closed bars) mmHg CO2 (pHe 7.4) for 10 min in the presence or absence of Gö6976 (1 µM, 30 min preincubation) or PMA (25 µM, 24 h preincubation). p-JNK and total JNK were determined by Western blot. Graph represents the p-JNK/JNK ratio, values are expressed as mean ± SEM, n = 3. **, p<0.01. Representative Western blots of p-JNK and total JNK are shown. (TIF) [file pone.0046696.s004.tif]
