# Supplementary material for: Evolutionary Conserved Role of c-Jun-N-Terminal Kinase in CO2-Induced Epithelial Dysfunction
Source: PLoS One. 2012 Oct 8;7(10):e46696. doi: 10.1371/journal.pone.0046696 (PMC3466313; doi:10.1371/journal.pone.0046696)
Supplement: Methods S1 — Supporting Methods. (PDF) [file pone.0046696.s005.pdf]

Evolutionary Conserved Role of c-Jun-N-terminal Kinase in CO_2-induced Epithelial Dysfunction

István Vadász, Laura A. Dada, Arturo Briva, Iiro Taneli Helenius, Kfir Sharabi, Lynn C. Welch, Aileen M. Kelly, Benno A. Grzesik, G. R. Scott Budinger, Jing Liu, Werner Seeger, Greg J. Beitel, Yosef Gruenbaum, and Jacob I. Sznajder

Supporting Information

Materials and Methods

Reagents

Cell culture reagents were from Mediatech Inc (Herndon, VA). $^{22}Na^{+}$ was from GE Healthcare (Piscataway, NJ) and ^{3}H-mannitol was from Perkin Elmer (Life Sciences, Inc, Boston, MA). The myristoylated PKC-ζ peptide inhibitor was from Biomol International (Plymouth Meeting, PA). Lipofectamine 2000 and Opti-MEM I reduced serum medium were from Invitrogen, (Carlsbad, CA). A monoclonal antibody directed against the Na,K-ATPase α_1 subunit (clone 464.6) was from Upstate Biotechnology (Lake Placid, NY; catalog number: 05-369). The a5 antibody against the *Drosophila* Na,K-ATPase was from the Developmental Studies Hybridoma Bank, University of Iowa). The antibody against Toll was from Santa Cruz Biotechnology (Santa Cruz, CA; catalog number: sc 33-741). Anti-p-JNK, -JNK and -p-c-Jun were from Cell Signaling (Danvers, MA; catalog number: #9251, #9252 and #9261, respectively). The antibody against JNK that was used in A549 cells was from BD Biosciences (San Jose, CA; catalog number: BD 554285) Secondary goat anti-mouse-horseradish peroxidase (HRP) was from Bio-Rad (Hercules, CA) and goat anti-rabbit-HRP was from Cell Signaling (Danvers, MA). All other chemicals were purchased from Calbiochem (San Diego, CA).

Animals

Pathogen-free adult male Sprague-Dawley rats were from Harlan (Indianapolis, IN). Animals were provided with food and water *ad libitum*, were maintained on a 12:12-h light-dark cycle.

Isolation and culture of alveolar epithelial cells

Alveolar epithelial type II cells were isolated from the lungs of Sprague-Dawley rats weighing 200-225 g, as previously described [1]. The day of isolation and plating was designated culture *day 0*. All experiments were conducted on *day 3*. A549 cells (ATCC CCL 185) were grown in DMEM supplemented with 10% fetal bovine serum, 100 U/ml penicillin, 100 μg/ml streptomycin. A549 cells expressing an empty vector or DN PKC-ζ were grown in the presence of G418 [2]. Experiments were performed on confluent monolayers of A549 cells. All cells were incubated in a humidified atmosphere of 5% CO_2-95% air at 37 °C.

CO_2 media

The composition of media for various CO_2 levels has been previously described [2,3]. Briefly, initial solutions were prepared with DMEM-Ham's F-12 medium-Tris base (3:1:0.5) containing 10% fetal bovine serum with 100 U/ml penicillin, and 100 μg/ml streptomycin. The buffering capacity of the media was modified by changing its initial pH with a Tris base in order to obtain a pH of 7.4 with the various CO_2 levels (pCO_2 of 40, 60, 80 and 120 mmHg). In some experiments, modeling extracellular acidosis, an initial pH of 6.8 was used, resulting in a final pH of 7.2 and a pCO_2 of 40 mmHg. Medium was placed overnight in a humidified chamber (C-174 Chamber, Biospherix, Ltd., Redfield, NY) to achieve the desired CO_2 levels and pH prior to starting the experiments.

Biotinylation of cell surface proteins

Cells were labeled for 20 min using 1 mg/ml EZ-link NHS-SS-biotin (Pierce Chemical Co., Rockford, IL) and surface proteins were pulled-down with streptavidin-Sepharose beads (Pierce Chemical Co., Rockford, IL) as described previously [4,5,6]. Proteins were analyzed by SDS-PAGE and Western blot analysis.

Western blot analysis

Protein concentration was quantified by Bradford assay (Bio-Rad, Hercules, CA) and proteins were resolved in 10%-12.5% polyacrylamide gels. Thereafter, proteins were transferred to nitrocellulose membranes (Optitran, Schleider & Schuell, Keene, NH) using a semi-dry transfer apparatus (Bio-Rad, Hercules, CA). Incubation with specific antibodies was performed overnight at 4 °C. Blots were developed with a chemiluminescence detection kit (PerkinElmer Life Sciences, Boston, MA) used as recommended by the manufacturer. The bands were quantified by densitometric scanning (Image J 1.29X, National Institutes of Health).

RNA knockdown in A549 cells

A specific siRNA against JNK_1 was obtained from Santa Cruz Biotechnology (Santa Cruz, CA). A549 cells (American Type Culture Collection; Manassas, VA) were plated in 60-mm cell culture dishes and transfected with 100 pmol siRNA or scrambled (scr) RNA using lipofectamine RNAiMax (Invitrogen, Carlsbad, CA) as recommended by the manufacturer. Experiments were performed 24 h later.

***In vitro* JNK kinase assay**

The in vitro kinase assay was performed as described previously [3,7]. To assess whether JNK_1 directly phosphorylated the Na,K-ATPase purified Na,K-ATPase α_1-subunit (Sigma-

Aldrich, St. Louis, MO) or an ATII whole cell lysate were incubated with activated JNK_1 (Cell Signaling, Danvers, MA) in the presence of 10 μCi [γ-^{32}P]–ATP and100 μM ATP for 30 min at 30°C in 35 mM Tris, pH 7.5, 10 mM $MgCl_2$, 5 mM EGTA, 1 mM $CaCl_2$, and 10 mM β-glycerolphosphate. The reactions were stopped by adding SDS sample buffer and boiling the samples. The proteins were separated by SDS-PAGE, and radio-labeled areas were detected by autoradiography.

Isolated-perfused rat lung model

The isolated lung preparation has been described in detail previously [2,3,8]. Briefly, the lungs and heart of anesthetized rats were removed en bloc. The pulmonary artery and left atrium were catheterized and perfused continuously with a solution of 3% bovine serum albumin (BSA) in buffered physiological salt solution (135.5 mM Na^+, 119.1 mM Cl^-, 25 mM HCO_3^-, 4.1mM K^+, 2.8 mM Mg^+, 2.5 mM Ca^{2+}, 0.8 mM SO_4^{2-}, 8.3 mM glucose). Trace amounts of FITC-albumin was also added to the perfusate. The recirculating volume of the constant pressure perfusion system was 90 ml; arterial and venous pressures were set at 12 and 0 cm H_2O respectively. The vascular pressures were recorded every 10 sec with a multichannel recorder (Cyber Sense Inc. Nicholasville, KY). The lungs were immersed in a "pleural" bath (100 ml) filled with the same BSA solution. The entire system was maintained at 37 °C in a water bath. Perfusate pH was maintained at 7.40 by bubbling with a gas mixture of 95%O2/5%CO2. The lungs were then instilled via the tracheal cannula in two sequential phases with a total of 5 ml volume of the BSA solution containing 0.1mg/ml EBD-albumin, 0.02 μCi/ml of $^{22}Na^+$ and 0.12 μCi/ml of ^{3}Hmannitol. Samples were taken from the instillate, perfusate, and bath solutions after an equilibration time of 10 minutes from the instillation and again 60 min later. To ensure a homogenous sampling of the instillate, a volume of 2 ml was aspirated and reintroduced into the airspaces three times before removing each sample. All samples were centrifuged at 3000 g for 10 minutes. Absorbance analysis of the supernatant or

EBD albumin was performed at 620 nm in a Hitachi model U2000 spectrometer (Hitachi, San Jose, CA). Analysis of FITC-albumin (excitation 487 nm and emission 520 nm) was performed in a Perkin-Elmer fluorometer (model LS-3B, Perkin-Elmer, Oakbrook, IL). Scintillation counts for $^{22}Na^{+}$ and ^{3}H-mannitol were measured in a Beckman beta counter (model LS 6500,Beckman Instruments Inc., Fullerton, CA).

Maintenance of *Drosophila* S2 cells and CO_2 exposure

Drosophila S2 cells were grown at room temperature in Schneider's insect medium containing 10% FBS (Valley Biomedical) and 0.2% Penicillin-Streptomycin (GIBCO). For cell attachment, plates were treated with 1N HCl for 1h, washed 3 times with sterile water, 0.5mg/mL Concanavalin A (Calbiochem) for 1h, washed once with sterile water, and S2 cells allowed to attach for 1hr. CO_2 treatments were performed at 15% CO_2 using pre-equilibrated media buffered to pH equal to air condition, with S2 cells attached to 6-well plates. Positive control DJNK (Basket) activation was achieved using soluble *E. coli* peptidoglycan (InvivoGen) at 25mg/ml final concentration for 15 min.

Maintenance of *C. elegans* strains and CO_2 exposure

C. elegans strains were handled as described previously [9].N2, *jnk-1(gk7)* and *kgb-2(gk361)* null mutations were obtained from the *C. elegans* Genetic Center. DYNAMENT CO_2 controller with a mini infrared sensor (0–20% CO_2) was connected to a sealed Perspex incubator. CO_2 was flowed to the incubator via the controller until reaching the desired level and was balanced using air.

References

1. Ridge KM, Olivera WG, Saldias F, Azzam Z, Horowitz S, et al. (2003) Alveolar type 1 cells express the alpha2 Na,K-ATPase, which contributes to lung liquid clearance. Circ Res 92: 453-460.
2. Briva A, Vadasz I, Lecuona E, Welch LC, Chen J, et al. (2007) High CO2 levels impair alveolar epithelial function independently of pH. PLoS ONE 2: e1238.
3. Vadasz I, Dada LA, Briva A, Trejo HE, Welch LC, et al. (2008) AMP-activated protein kinase regulates CO2-induced alveolar epithelial dysfunction in rats and human cells by promoting Na,K-ATPase endocytosis. J Clin Invest 118: 752-762.
4. Dada LA, Chandel NS, Ridge KM, Pedemonte C, Bertorello AM, et al. (2003) Hypoxia-induced endocytosis of Na,K-ATPase in alveolar epithelial cells is mediated by mitochondrial reactive oxygen species and PKC-zeta. J Clin Invest 111: 1057-1064.
5. Lecuona E, Ridge K, Pesce L, Batlle D, Sznajder JI (2003) The GTP-binding protein RhoA mediates Na,K-ATPase exocytosis in alveolar epithelial cells. Mol Biol Cell 14: 3888-3897.
6. Lecuona E, Dada LA, Sun H, Butti ML, Zhou G, et al. (2006) Na,K-ATPase alpha1-subunit dephosphorylation by protein phosphatase 2A is necessary for its recruitment to the plasma membrane. FASEB J 20: 2618-2620.
7. Soberanes S, Urich D, Baker CM, Burgess Z, Chiarella SE, et al. (2009) Mitochondrial complex III-generated oxidants activate ASK1 and JNK to induce alveolar epithelial cell death following exposure to particulate matter air pollution. J Biol Chem 284: 2176-2186.
8. Saldias FJ, Azzam ZS, Ridge KM, Yeldandi A, Rutschman DH, et al. (2001) Alveolar fluid reabsorption is impaired by increased left atrial pressures in rats. Am J Physiol Lung Cell Mol Physiol 281: L591-597.

9. Sharabi K, Hurwitz A, Simon AJ, Beitel GJ, Morimoto RI, et al. (2009) Elevated CO2 levels affect development, motility, and fertility and extend life span in Caenorhabditis elegans. Proc Natl Acad Sci U S A 106: 4024-4029.
